# Genetic and Genomic Landscape of Secondary and Therapy-Related Acute Myeloid Leukemia

**DOI:** 10.3390/genes11070749

**Published:** 2020-07-06

**Authors:** Alexandra Higgins, Mithun Vinod Shah

**Affiliations:** Division of Hematology, Mayo Clinic, Rochester, MN 55905, USA; higgins.alexandra@mayo.edu

**Keywords:** acute myeloid leukemia, myelodysplastic syndrome, myeloproliferative neoplasm, next-generation sequencing, molecular markers, clonal hematopoiesis, allogeneic transplant

## Abstract

A subset of acute myeloid leukemia (AML) arises either from an antecedent myeloid malignancy (secondary AML, sAML) or as a complication of DNA-damaging therapy for other cancers (therapy-related myeloid neoplasm, t-MN). These secondary leukemias have unique biological and clinical features that distinguish them from de novo AML. Over the last decade, molecular techniques have unraveled the complex subclonal architecture of sAML and t-MN. In this review, we compare and contrast biological and clinical features of de novo AML with sAML and t-MN. We discuss the role of genetic mutations, including those involved in RNA splicing, epigenetic modification, tumor suppression, transcription regulation, and cell signaling, in the pathogenesis of secondary leukemia. We also discuss clonal hematopoiesis in otherwise healthy individuals, as well as in the context of another malignancy, and how it challenges the conventional notion of sAML/t-MN. We conclude by summarizing the current and emerging treatment strategies, including allogenic transplant, in these complex scenarios.

## 1. Introduction

**Figure 1:**
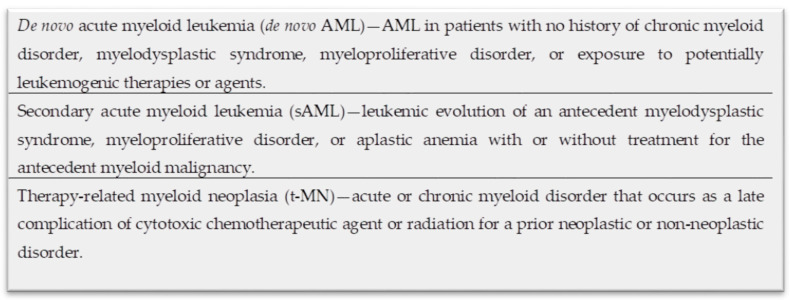
Definition of key terms.

Acute myeloid leukemia (AML) can arise de novo or as a complication of a prior malignancy. Secondary leukemia can be classified into one of two broad entities: AML arising from an antecedent myeloid malignancy—myelodysplastic syndrome (MDS), myeloproliferative neoplasm (MPN), or MDS/MPN overlap syndrome, where progression to AML is considered a part of the natural history of the disease (secondary AML (sAML))—as well as therapy-related myeloid neoplasm (t-MN), which arises as a complication of prior cytotoxic therapy (Figure 1). When combined, these entities account for 25–35% of all AML cases [1,2]. The incidence of sAML and t-MN is rising, and is likely related to the increasing survivorship of prior solid and hematological malignancies [3], increasing use of chemotherapy and other treatment modalities in the adjuvant setting [4], and improved reporting of myeloid malignancies [3,5]. Secondary AML and t-MN are characterized by unique cytogenetic and molecular abnormalities that confer distinct clinical and biological features. These are consistently associated with poor prognosis [2,6,7,8,9,10], with age and adverse cytogenetic features predicting a poor response to treatment [10,11]. Determining the secondary status of AML has significant therapeutic implications as well. Recently, CPX-351 (liposomal formulation of cytarabine and daunorubicin) was approved for use in high-risk AML including AML with myelodysplasia-related changes (AML-MRC) and t-AML. After remission is achieved, patients with sAML or t-MN are recommended to undergo allogeneic stem cell transplant, though there is a significant debate in the field regarding whether transplant improves outcomes in many of these patients.

Traditionally, sAML/t-MN have been either lumped with de novo AML or excluded from trials, precluding an analysis of secondary leukemia as a unique entity. With unprecedented advances in molecular genetics, the clonal hierarchy of secondary leukemia is being elucidated. A deeper insight into the biology has opened doors to individualized prognostication as well as the development of therapeutic approaches in the hope of achieving durable remissions and improving survival. The aim of this review is to compare and contrast the clinical and biological features of de novo AML with sAML arising from MDS, MDS/MPN overlap syndromes, or MPN in blast phase (MPN-BP) and t-MN. Finally, we discuss the emerging field of clonal hematopoiesis (CH) that precedes a majority of cases of AML by years or even decades, and thus, poses an interesting dilemma as to how AML is classified. We conclude that further understanding of the genomic landscape of sAML and t-MN is essential to better understand the prognostic impact of the molecular characteristics of the disease on progression to acute leukemia and how these differences might be important for determining most effective management of these complex diseases.

## 2. Secondary AML Arising from Myelodysplastic Syndrome (MDS) and MPN in Blast Phase (MPN-BP)

The 2016 World Health Organization (WHO) classification of myeloid neoplasms and acute leukemia includes two categories for secondary leukemias: AML-MRC (Table 1) and t-MN. The median time to development of sAML from MDS or chronic myelomonocytic leukemia (CMML) is around 18 months while the median time is 43 months from an antecedent MPN. AML arising from an antecedent MPN or CMML has been demonstrated to have a poorer response to chemotherapy and a worse overall survival (OS) than AML from antecedent MDS, while all entities have a poor prognosis compared to de novo AML, independent of cytogenetics and age [1].

MDS is a clonal disease of the hematopoietic stem cells (HSCs) characterized by ineffective hematopoiesis and increased apoptosis, which results in cytopenia [12,13,14,15]. Leukemic progression from MDS occurs in about 20% of patients with a range from 2% in refractory cytopenia with unilineage dysplasia to 40% in MDS with excess blasts [16]. Approximately 11% of cases of AML are thought to have progressed from an antecedent MDS [1]. A combination of clinical and cytogenetic variables, known as the revised international prognostic scoring system (R-IPSS), is the most commonly used clinical tool to estimate the risk of progression to sAML [17]. Poor prognosis is associated with −7, inv(3)/t(3q)/del(3q), double clone including −7/del(7q), or complex karyotype (CK) with ≥3 abnormalities, which substantially increases the risk of leukemic progression [17,18]. For instance, the presence of CK increases the risk of leukemic transformation by approximately fivefold. Since then, multiple attempts to refine the tool incorporating molecular abnormalities have been proposed [19,20].

**Table 1:** Per WHO classification, cytogenetic abnormalities sufficient to diagnose acute myeloid leukemia (AML) with myelodysplasia-related changes when ≥20% peripheral blood or bone marrow blasts are present and prior therapy has been excluded [21].

Complex Karyotype (CK)	Unbalanced Abnormalities	Balanced Abnormalities
3 or more abnormalities	−7/del(7q)	t(11;16)(q23.3;p13.3)
	del(5q)/t(5q)	t(3;21)(q26.2;q22.1)
	i(17q)/t(17p)	t(1;3)(p36.3;q21.2)
	-13/del(13q)	t(2;11)(p21;q23.3)
	del(11q)	t(5;12)(q32;p13.2)
	del(12p)/t(12p)	t(5;7)(q32;q11.2)
	idic(X)(q13)	t(5;17)(q32;p13.2)
		t(5;10)(q32;q21.2)
		t(3;5)(q25.3;q35.1)

Classical MPNs (*BCR-ABL*-negative MPNs) include polycythemia vera (PV), essential thrombocythemia (ET), and primary myelofibrosis (PMF) [21]. These diseases exist on a continuum as both PV and ET may progress to secondary myelofibrosis and all entities can progress to MPN-BP [22]. For the majority of cases, the development of these MPNs is driven by one of three mutations including Janus Kinase 2 gene (*JAK2*), calreticulin (*CALR*), or myeloproliferative leukemia protein (*MPL*); however, additional driver mutations contribute to profound heterogeneity among these neoplasms [23,24,25,26,27,28]. As with other myeloid malignancies, there is significant interest in identifying risk factors predicting the progression to the blast phase. The estimated risk of leukemic transformation differs significantly among the three entities—4% for ET, 7% for PV, and 14% for PMF at 20 years [29,30,31]. Apart from morphology, high circulating CD34 counts, concurrent cytopenia, and prior alkylating therapy—albeit controversial—have been suggested to predict the risk of leukemic progression [32]. Among the genetic factors, unfavorable karyotype, as defined in the dynamic international prognostic scoring system (DIPSS)-plus, signifies a six-fold higher risk of progression to MPN-BP [33]. Among the driver mutations, *MPL* mutated MF carries a nine-fold higher a risk of progression to MPN-BP compared to *JAK2*-heterozygous disease [34]. Interestingly, *CALR*-mutated PMF carries lesser risk of leukemic transformation compared to *JAK2-* and *MPL*-mutated PMF [35].

Many of the additional somatic mutations found in MPNs have been discovered to be present at diagnosis and not newly acquired at the time of leukemic transformation [24]; therefore, a comprehensive mutation screening at diagnosis can offer considerable prognostic information regarding the risk of leukemic progression [23,34,36]. Recently, using a large cohort of chronic phase MPN patients and 63 clinical and genomic variables, eight genomic subgroups with distinct phenotypes, including the risk of leukemic transformation, were identified. For the ease of use in clinical practice, a tool capable of generating personally tailored predictions of clinical outcomes is available (https://cancer.sanger.ac.uk/mpn-multistage/) [34].

As with other myeloid neoplasms, heterozygous *TP53*-mutated clones may rarely be found early in the disease process of MPNs, whereas the loss of the wild-type allele is associated with a 15-fold higher risk of leukemic progression [37,38]. The amplification of chromosome 1q32—leading to gains in the function of the *MDM4* gene, which, in turn, inhibit *TP53*-mediated transcriptional transactivation—represents another mechanism of *TP53*-pathway dysfunction leading to MPN-BP. Chromosome 1q32 amplifications are seen in 20% of MPN-BP compared to 0.32% of the chronic phase [37,39]. Taken together, 45% of cases of MPN-BP harbor a *TP53*-related defect either by *TP53* gene mutations/haploinsufficiency or amplifications of chromosome 1q [37].

Chromosomal aberrations are seen in >60% of patients with MPN. An increased number of abnormalities is significantly associated with age, progression, and leukemic transformation. Aberrations of chromosome 1q and 9p are associated with progression to secondary myelofibrosis and the accelerated phase. Chromosome abnormalities associated with MPN-BP include 1q and 3q amplifications, deletions of 7q, 5q, 6p, 7p (19% vs. 0.5% in the chronic phase) [40] and uniparental disomies (UPDs) of 19q and 22q [39]. The genes associated with these chromosome aberrations include *MDM4* (1q), *IKZF1* (7p), *EZH2* (7q), *CUX1* (7q), and *JARID2* (6p) [41].

A special consideration, and a topic of frequent debate, is the contribution of treatment of MPN to subsequent development of MPN-BP. A large Swedish registry study of >11,000 patients found that the use of hydroxyurea was not associated with an increased risk of leukemic progression [42]. The same study observed that use of busulfan (>1 g) or radioactive phosphorus (^32^P, >1000 MBq) was associated with an increased risk of progression to AML or MDS (4.6 and 3.4-fold, respectively). Exposure to more than one cytoreductive treatment was also associated with an increased risk of progression to AML; however, these patients likely have a more aggressive biology, requiring the escalation of treatment, and may have progressed regardless of cytoreductive treatment [43]. Twenty-five percent of patients with leukemic progression were not exposed to any cytoreductive therapy, suggesting a clear role of underlying factors that are not related to prior treatment.

## 3. Mutational Landscape of Secondary AML Arising from Antecedent Myeloid Neoplasms

The development of most myeloid malignancies is driven by somatic mutations resulting in the clonal expansion of HSCs. Subsequent acquired mutations may either be relatively inconsequential passenger mutations or driver mutations which contribute to clonal expansion and disease progression. Many of these mutations have been identified using high-resolution genome analysis such as microarrays and next-generation sequencing (NGS). In recent years, there has been an explosion of studies attempting to provide a framework of the genetic diversity observed in sAML and t-MN. These studies have confirmed the distinct mutational signatures of de novo, sAML, and t-MN. On the other hand, there is a significant overlap in the genomic landscape of the sAML arising from antecedent MDS, MPN, and MDS/MPN overlap syndromes. Therefore, we discuss the biology of leukemic transformation from antecedent myeloid neoplasm as a group in the following sections.

Targeted mutational analysis in strictly defined sAML showed that the most frequently mutated genes were those involved in RNA splicing (55%), DNA methylation (46%), chromatin modification (42%), RAS signaling (42%), transcriptional regulation (34%), and the cohesin complex (22%) (Table 2) [10]. The presence of a mutation in one of the following eight genes, seen frequently in MDS and termed ‘secondary-type mutations’, is >95% specific for sAML: serine and arginine-rich splicing factor-2 (*SRSF2*), splicing factor-3B, subunit 1 (*SF3B1*), U2 small nuclear RNA auxiliary factor-1 (*U2AF1*), zinc finger CCCH-type, RNA binding motif and serine/arginine-rich-2 (*ZRSR2*), additional sex combs-like-1 (*ASXL1*), enhancer of Zeste-2 polycomb repressive complex subunit-2 (*EZH2*), B-cell lymphoma-6 (*BCL-6*) corepressor (*BCOR*), or stromal antigen-2 (*STAG2*). In contrast, nucleophosmin-1 (*NPM1*) mutations, *MLL*/11q23 rearrangements, and core-binding factor rearrangements are >95% specific for de novo AML.

Mutations in transcription factors and signal transduction genes are suspected to be important for leukemic transformation. Early paired analyses of MDS and sAML samples show that leukemic progression is characterized by acquisition of new mutations, while the preexisting mutations are carried forward—thus resulting in subclones that contain increasing numbers of mutations. The accumulation of somatic mutations, including activating mutations of tyrosine kinases and loss-of-function mutations of hematopoietic transcription factors, has been implicated as the mechanism of progression from MDS to sAML [16,44]; however, some researchers propose that a single critical event/mutation drives the progression, since the risk of progression was found to be fairly constant following diagnosis [45]. An additional theory of the clonal evolution of MDS to sAML is clone sweeping, during which a new or preexisting subclone outcompetes other existing subclones, eventually populating the entire hematopoietic compartment [46]. A similar paired analysis of transformed MPN-BP showed that mutations in chromatin modification (*ASXL1*, *EZH2*), RNA splicing (*SRSF2*), and signaling pathways (*CBL*, *NF1*, *FLT3*, *RAS*) are enriched in the blast phase [36]. Table 3 summarizes the acquired mutations implicated in progression from MDS to AML and Table 4 summarizes the acquired mutations implicated in MPN-BP with the hazard ratio for leukemic progression when available.

### 3.1. Mutations in Epigenetic Regulators

The broad category of epigenetic regulators includes genes involved in DNA methylation and those involved in chromatin remodeling. Mutations in epigenetic regulators impair myelopoiesis, likely through abnormal DNA hypermethylation. Mutations of the *TET2* gene, which encodes a 2-oxoglutarate/Fe^2+^ oxygenase which catalyzes the conversion of methylcytosine to hydroxymethylcytosine, are frequently mutated in myeloid malignancies including 20–25% of MDS and 12–15% of MPN [27]. *TET2* mutations have an unclear role in the progression to MPN-BP. For example, one study found deletions of 4q (*TET2* mutations) were evenly distributed between the chronic phase and MPN-BP [39], while others showed a significant association of *TET2* mutations with an increased risk of leukemic progression [24,34].

Mutations in isocitrate dehydrogenase (*IDH*)*-1* and *-2* are less frequent in MDS. Combined, they are present in 12% of MDS—4%, 12%, 14%, 14%, and 23% in refractory anemia with ring sideroblasts, refractory cytopenia with multilineage dysplasia, MDS with excess blasts (MDS-EB)-1 and MDS-EB-2, respectively. There is conflicting evidence regarding its impact on progression to sAML [50,66], with some studies reporting a sevenfold increased risk of progression to sAML with *IDH1* mutations only [49,67], while others support the increased risk of progression with either mutation [50]. Similarly, *IDH* mutations are uncommon (1–4%) in chronic phase MPN, but are enriched in MPN-BP (up to 30%) [62,63]. The risk of leukemic transformation is context dependent—in PMF, the presence of *IDH-1* or *-2* increases risk by three- to six-fold; in PV, the risk is up to 55-fold higher, while in ET, *IDH* mutation status was not contributory to leukemia free survival [26,32,34,50].

Loss-of-function (LOF) mutations in *EZH2*, located on 7p, are found in 6% of patients with PMF and are associated with a significantly reduced leukemia free survival (LFS) and OS. It is suspected that the *EZH2* mutation is acquired early in the disease process, as prospective studies of PMF patients have shown that late acquisition of an *EZH2* mutation is exceedingly rare [68]. These LOF mutations are found more frequently in PMF (25%) compared to PV and ET (1–3%), [54] and were originally described in MDS and MDS/MPN overlap syndromes [69].

*ASXL1* encodes for an epigenetic regulator involved in the process of chromatin remodeling and mutation in this gene has been found to have a poor prognosis across myeloid malignancies [25,70]. Mouse models with Asxl1 deletion have shown progressive and profound cytopenia with dysplasia and the inability of HSCs to regenerate and differentiate [71]. *ASXL1* mutations are common in MDS (15-20%), PMF, and secondary MF (36%), but are rare in PV (1%) and ET (not detected) [69,71,72]. Frameshift mutations of *ASXL1* have been associated with a 2.4-fold increased risk of leukemic progression [47], while the role of point mutations is not fully elucidated. Concurrent mutations in *SETBP1* drive leukemic progression by augmenting the *ASXL1* mutant differentiation block and inhibiting apoptosis, while driving increased self-renewal [73].

### 3.2. Mutations in the Transcriptional Regulator Genes

Mutations of transcription factors such as *RUNX1*, *ETV6*, *IKZF1*, *CUX1*, *TP53*, and *PHF6* are commonly implicated in leukemic progression across chronic myeloid malignancies. *RUNX1* is one of the most frequently mutated genes (11–13%) in MDS and RUNX1 mutations are enriched by threefold in those progressed to sAML. The acquisition of a mutation in *MLL*-PTD or *FLT3*-ITD in the context of mutated *RUNX1* strongly drives leukemic transformation [16]. The role of *RUNX1* mutations in leukemic progression has been supported in further functional studies [74].

In addition to *EZH2,* discussed above, another gene of interest located at 7p is IKAROS family zinc finger-1 (*IKZF1*). An evaluation of paired samples shows that mutations of *IKZF1* are acquired late in disease. The mechanism by which *IKZF1* loss contributes to leukemic transformation is not clear; however, putative mechanisms include increased chromosomal instability in the HSCs or augmentation of JAK-STAT signaling. *IKZF1* deletions are common in de novo acute lymphoblastic leukemia (ALL) [75], but have not been described in de novo AML.

DNA damage response regulator tumor protein 53 (*TP53*) and its negative regulator protein phosphatase Mn^2+/^Mg^2+^-dependent 1D (*PPM1D*) are discussed in the ‘Biology of Therapy-Related Myeloid Neoplasm’ section.

### 3.3. Mutations in the RNA Splicing Factors (SF)

SF genes encode for components of the spliceosomes which include small nuclear RNAs and proteins that catalyze the splicing reaction – removing the non-coding sequences (introns) from precursor messenger RNA and ligating exons in order to form a mature messenger RNA. SF genes are the most common molecular abnormalities in MDS, accounting for up to 64% cases, with just four genes—*SF3B1*, *SRSF2*, *U2AF1*, and *ZRSR2*—being the most commonly mutated [76]. SF gene mutations are frequently identified in myeloid disorders with underlying dysplasia [10,65] including MDS and MDS/MPN overlap syndromes. *SRSF2* mutations are seen in 15% of MDS cases and predict a fourfold increased risk of sAML [50]. *U2AF1* are less frequent (5–8%), but are found threefold more frequently in those with sAML [77].

### 3.4. Mutations in the Signaling Pathways

Finally, mutations in proliferative genes in the tyrosine kinase and RAS pathways occur late and are indicative of impending transformation to sAML [78]. While the relative risk of each mutation for progression to leukemia is not clearly established, mutations in NRAS, KRAS, and fms-like tyrosine kinase-3 (FLT3) was seen in approximately 40% of MDS patients at the time of transformation [10] and, when found, patients have a shorter time to transformation and a shorter survival [51,79]. The constitutive activation of the FLT3-mediated signaling leads to the activation of the downstream STAT- and RAS/RAF/MEK/ERK pathways, which in turn, leads to an uncontrolled, growth factor-independent proliferation of the HSCs.

### 3.5. Genetic Risk Factors for Leukemic Progression from Chronic Myelomonocytic Leukemia

MDS/MPN overlap syndromes include CMML, atypical chronic myeloid leukemia (aCML) *BCR-ABL1*-, unclassifiable MDS/MPN, and MDS/MPN with ring sideroblasts and thrombocytosis [21]. These are complex disorders that exhibit an overlapping phenotype with both dysplastic and proliferative features. Evidence regarding the progression to sAML is sparse among these more recently described entities due to their rarity. CMML has historically been studied in conjunction with either MDS or MPN instead of as a distinct MDS/MPN overlap syndrome and this limits available data regarding the biology of the unique disease entity [21]. Interestingly, the karyotype is normal in 70% of CMML patients; however, there are a high number of molecular mutations with 5–20 per case [80,81]. Low risk karyotypes include those that are normal or those that harbor sole –Y or der (3q). High risk karyotypes include those that are complex or monosomal and intermediate risk includes those not otherwise categorized as low or high risk [82,83].

As with MDS and MPN, mutations in *TET2*, *ASXL1,* and *SRSF2* are common in CMML [80,84,85] with an incidence of 60%, 50%, and 40%, respectively [85,86,87]. Lower frequency mutations include *SETBP1*, *NRAS*/*KRAS*, *CBL*, and *EZH2* [81,88] with cell signaling mutations (*RAS*, *CBL*) seen in around 30% of cases. *TET2* and *SRSF2*, although common, are not thought to promote leukemic progression [85,89]. Mutations in *ASXL1* and *RUNX1* (30%) have been implicated in driving disease to sAML and are frequently found to co-exist. *ASXL1* is a predictor of aggressive behavior with proliferative phenotype [84,90] and has been incorporated into prognostic scoring systems predicting progression to sAML [74,86,91]. It should be emphasized that the impact of *ASXL1* is context dependent. For example, the co-expression of *RUNX1* and *ASXL1* mutants increases myeloid stem cells by blocking differentiation and increasing self-renewal activity through transcriptional activations of hypoxia-inducible factor 1 (HIF1-α) suggesting that these mutant genes cooperate to promote leukemic transformation [86]. On the other hand, harboring a mutant *TET2* partially offsets the poor prognostic impact of the mutant *ASXL1*, though mechanism for such an occurrence is not clear [85,90].

*TP53* mutations are uncommon in CMML (<5%). When found, it is present at diagnosis without co-occurring *TET2* and/or *ASXL1* mutations suggesting a unique pathogenesis [92]. Moreover, different from MDS or MPN, *TP53* mutations in CMML have not been shown to be associated with CK [92] and its role in leukemic progression is unclear.

## 4. Mutational Landscape of Therapy-Related Myeloid Neoplasms

Therapy-related myeloid neoplasms have been defined by the WHO as myeloid neoplasms, including the spectrum of MDS, AML, and MDS/MPN overlap syndromes, that occur any time after exposure to DNA damaging agents [21]. Due to differing definitions, the exact incidence of t-MN is unclear. For example, one study estimated that t-MN account for 10–20% of all AML, MDS, and MDS/MPN, while a large Surveillance, Epidemiology, and End Results (SEER) study of AML only reported 6.5% of patients with prior chemotherapy and/or radiation [1]. Alkylating agents (platinum compounds, busulfan, cyclophosphamide, chlorambucil, melphalan etc.), topoisomerase II inhibitors (anthracyclines, etoposide, etc.), and nucleoside analogues (fludarabine), are all established causes of t-MNs [93,94,95,96,97] while many other therapeutics (lenalidomide, poly (ADP ribose) polymerase inhibitors, peptide receptor radionuclide therapy) have also been implicated [98,99,100]. The leukemogenic effects of radiation are clearly illustrated with the high incidence of myeloid malignancies in survivors of the atomic bomb explosions in 1945 with a peak incidence at 5–7 years after exposure [101]. A large registry study found that the median latency time from cytotoxic therapy to development of t-AML was 63 months [1], while another showed that median time to develop t-AML was a little shorter for t-AML than t-MDS (44 vs. 54 months) [102]. However, acknowledging the commonality of prior cytotoxic exposure and overall comparable clinical course, the 2016 WHO classification does not distinguish between t-MDS and t-AML.

Two classic patterns of development of t-AML have been described. The first is onset of t-AML 5-7 years after alkylating chemotherapy or radiation exposure. The second pattern is that caused by topoisomerase II inhibitors such as etoposide and anthracyclines leading to balanced translocations such as those involving the lycine methyltransferase-2A (*KMT2A*) or mixed-lineage leukemia (*MLL*) gene at 11q23 with a latency period of 2–3 years [103]. t-MN has also been described after autologous transplant with a significant association with prior fludarabine exposure and poor survival [104].

Combined cytogenetic and targeted genetic analysis of a large cohort of AML patients that included de novo AML, sAML, and t-MN [105] confirmed that t-MN are more likely to harbor cytogenetic aberrations (−5/del (5q), −7/del (7q), and/or CK) compared to de novo AML; however, the prevalence of these abnormalities was similar in sAML. Therapy-related MN is characterized by a higher frequency of *DNMT3A*, *FLT3*, *NPM1*, and *NRAS* mutations and significantly fewer mutations in the ‘secondary-type mutations’ such as *ASXL1*, *BCOR*, *RUNX1*, and *SRSF2* as compared to sAML.

Overall, t-MN has significant genomic heterogeneity, which is affected by type of cytotoxic exposure, age of patient, and even the presence of clonal hematopoiesis (CH) prior to toxic exposure. There have been no identified genetic patterns in t-MN besides the known association between topoisomerase-2 inhibitor exposures and *MLL* rearrangements [103], complex karyotypes and unbalanced loss of genetic material (particularly of chromosomes 5 and/or 7) associated with alkylating agents and radiation [105], the high frequency of *TP53* mutations, and, more recently, the enrichment of *PPM1D* mutations. *TP53* is the most widely studied gene in cancer in general and t-MN specifically. It is the most commonly mutated gene in t-MN and seen at a much higher frequency than de novo AML (16% vs. 8%). *TP53* mutations are repeatedly shown to be associated with worse overall outcomes in myeloid malignancies [58].

## 5. Biology of Therapy-Related Myeloid Neoplasm

As with other myeloid neoplasms, our understanding of t-MN has undergone a revolution with the wide adaptation of gene sequencing technologies. Whole-genome sequencing (WGS) of t-MN patients rather unexpectedly found that the number of single nucleotide variants (SNV) and transversions were similar between t-MN and de novo AML suggesting the cytotoxic chemotherapy does not induce genome-wide DNA damage [106,107] as previously hypothesized. In fact, in many cases, the *TP53* mutation is present for years before the development of t-MN, in two cases even predating the chemotherapy exposure. Healthy elderly controls are also found to have circulating *TP53*-mutated clones, supporting the notion that HSCs accumulate coding mutations as a function of age [106]. Mutated *TP53*, therefore, is often ancestral in t-MN, acquired before other molecular anomalies. There are multiple mechanisms by which mutations in *TP53* are thought to contribute to clonal expansion of emerging cancer cells. The *TP53* mutation is postulated to confer a selective growth advantage after exposure to cytotoxic chemotherapy or radiation. A mechanism different from the classical *TP53*-mediated DNA damage response, is the role of *TP53* in cell competition which selects for “least damaged cells” as elegantly described by Bondar and Medzhitov in 2010 [108]. Two types of activities of *TP53* are described: *TP53*-dependent apoptosis (or cell cycle arrest) occurring when a threshold of DNA damage is reached. The second activity emerges when DNA damage occurs, but the threshold for apoptosis does not occur. In this setting, cells with lower level *TP53* activity out-compete cells with higher level of *TP53* activity, and thus allow for competitive advantage of cells with reduced p53 activity [108]. This cell competition is demonstrated to occur at the level of HSCs and provides a mechanism for which *TP53*-mutated cells have selective advantage during cytotoxic therapies. Taken together, these findings suggest that *TP53* mutations are enriched in t-MN compared to de novo AML, and that this mutation may predate chemotherapy exposure and confer selective growth advantage to the cells harboring the mutated clone.

In non-*TP53*-mutated t-MN, it can be hypothesized that there is a similar age-related or ancestral mutation which confers selective growth advantage upon exposure to cytotoxic therapy and further accumulation of mutations leading to clonal expansion and t-MN. Numerous studies have demonstrated that *PPM1D* is a DNA-damage response regulator that is a part of the regulatory feedback loop for *TP53*. Activated *TP53* induces *PPM1D* leading to dephosphorylation of *TP53* and down-regulated apoptosis [109]. Truncating mutations of *PPM1D* are gain-of-function. Mutant *PPM1D* cells have more stable protein structures and decreased rate of apoptosis during subsequent exposure to DNA damaging chemotherapies leading to a selective clonal advantage [110]. *PPM1D* mutations are seen in a fascinating diversity of malignancies and pre-malignant states—including clonal hematopoiesis of indeterminate potential (CHIP), solid malignancies [111], and up to 20% of t-MN [10,110]—but are rarely mutated in de novo AML. Unlike *TP53* mutations, *PPM1D* mutations are not associated with complex cytogenetics or abnormalities in chromosome 5/7. *PPM1D* mutations are associated with previous platinum and etoposide exposure, but not with radiation exposure [110,112]. It is yet to be determined whether *PPM1D* mutations are drivers for leukemia development or simply a passenger sub-clone given the low variant allele frequencies that have been reported [10]. It is also notable that *TP53* and *PPM1D* mutations are among the more frequently mutated genes in patients with CHIP and both genes that are necessary for proper DNA repair. The detection of mutations in either of these genes in patients scheduled to receive cytotoxic chemotherapy may identify those patients at highest risk for chemo-refractory t-MN [111,113,114]. A summary of the acquired mutations implicated in pathogenesis of t-MN is available in Table 5.

An often-overlooked subset of patients at increased risk for t-MN are those carrying germline mutations in the genes that confer susceptibility to multiple malignancies, similar to the mechanism of Li-Fraumeni syndrome or dyskeratosis congenita. It is not possible to distinguish between t-MN and such a genetic predisposition without identifying the underlying germline mutation. A study of 47 breast cancer survivors who developed t-MN showed that 20% harbored a germline mutation in *BRCA1/2*, *TP53*, *PALB2*, or *CHEK2* [115]. These genes all play key roles in DNA repair pathways; therefore, these patients may have developed ‘de novo AML’ related to the germline mutations, although one could speculate on the role of these mutations in selective growth advantage when HSCs are exposed to cytotoxic therapies.

**Table 5:** Comparison of cytogenetic and molecular abnormalities in therapy-related myeloid neoplasm (t-MN) with de novo AML.

Functional Group	Genetic Abnormality	Frequency in de novo AML (%)	Frequency in t-MN (%)	Reference
Cytogenetics	Del(5q)	5–16	42	[116,117,118,119]
	Del7(q)/-7	4–14	49	
	Del 17p/-17	4	20	
	CK	5–17	48	
	Diploid karyotype	41–48	8	
Epigenetic regulation	*ASXL1*	10	4	[105,116,120]
	*DNMT3A*	30	20	
	*TET2*	17	10	
	*IDH1*	8–10	3–5	
	*IDH2*	9–10	0–5	
Signaling pathway	*FLT3*	24–28	8–16	
	*KIT*	4–6	0–3	
Splicing factor	*SF3B1*	10	3	
DNA damage response	*TP53*	2–12	13–37	
	*CEBPA*	9	3	
	*NPM1*	34	18	

Abbreviations: acute myeloid leukemia (AML); therapy-related myeloid neoplasms (t-MN); complex karyotype (CK).

## 6. The Role of Clonal Hematopoiesis in the Development of Myeloid Malignancies

An area of immense interest is the novel entity of CHIP, defined as the presence of somatic clonal mutations in patients who do not meet diagnostic criteria for a myeloid neoplasm. Since two-thirds of patients with AML are noted to have CH predating diagnosis by years or decades [121], it is unclear whether the leukemia that develops in the setting of CHIP is a distinct entity from de novo AML. Genovese et al. applied WGS to 12,380 study participants and found that CHIP was present in 10% of participants >65 years, while infrequently observed (1%) in patients <50 years [113]. Further follow-up of these patients found up to a 13-fold higher risk of a subsequent hematologic malignancy, suggesting that CHIP mutations may be initiating clonal events. On the other hand, using extremely sensitive sequencing technologies, 97% of patients had AML-related mutations detected up to 22 years predating the diagnosis of AML [122]. Further complicating the matter is the observation that up to 95% of 50–65-year-old adults harbor similar mutations without developing hematological malignancy [123]. Recent mathematical modeling of CH showed that genetic diversity of cells in the blood is predominantly determined by positive selection, rather than neutral genetic drift. The mutations that confer greater ‘fitness’ were associated with a higher risk of progression to AML [124].

Coexistent CH, the presence of CH in the context of a non-myeloid malignancy, is a phenomenon that is gaining attention. In a study of lymphoma patients undergoing autologous stem cell transplant, approximately 30% had a coexistent CH. The presence of CH predicted a 3.3-fold (14.1% vs. 4.3% at 10-years) risk of t-MN as well as a higher risk of death from cardiovascular diseases compared to those without CH [125]. A similar pattern was observed in the setting of diverse solid malignancies – the presence of CH in presumptive leukemia driver genes (CH-PD) was associated with both a shorter survival and a subsequent increase in hematological neoplasms. This finding, however, is not universal—a similarly designed study in multiple myeloma, as well as a population-based study of lymphoma patients undergoing transplant, did not confirm these findings [126,127].

This phenotypic diversity is, at least in part, due to a wide variety of timing of the ‘baseline’ sample obtained, genes interrogated, and variance allele frequency (VAF) threshold used. As high-throughput sequencing becomes mainstream, the next frontier would be to distill knowledge from the plethora of information available. At the minimum, standardization of the sequencing and bioinformatics techniques, as well as gene panels interrogated, will be required and large-scale prospective studies will be needed to estimate the risk of each mutation.

It is also clear that while genetics is pivotal in determining the risk of subsequent malignancies and mortality, there is a substantial contribution of non-genetic factors including co-existent mutation status, bone marrow microenvironment, immune editing, epigenetics, and extrinsic factors such as chemotherapy and/or radiation [116]. For example, a recent study of newly diagnosed MM patients elegantly showed the interplay of genetic risk factors with dysregulated immune surveillance: patients that had MDS-associated phenotypic aberrations (MDS-PA) also exhibited dysregulated immune surveillance in the form of reduced frequency of naive γδ T-cells and expansion of CCR7 ^negative^ regulatory T-cells [128]. Historically, the focus of study was primarily on the HSCs. However, conclusive evidence suggests that t-MN is driven by the synergistic effects on HSCs and the bone marrow microenvironment [129].

Another example of the impact of the interplay between genetic and extrinsic factors, Takahashi et al. demonstrated that the presence of CH significantly increases the risk of subsequent t-MN by 14-fold [130]. Similarly, Gillis et al. showed that elderly patients who developed t-MN were more likely to have CHIP [131]. Immunophenotyping complements genotypic approach in predicting the risk of t-MN. For example, the presence of CH (5.9-fold) or an aberrant expression of CD7 (6.6-fold), predicted an increased risk of t-MN, while all the patients with both CH and aberrant CD7 expression developed t-MN [114].

## 7. Treatment of Secondary Acute Myeloid Leukemia and Therapy-Related Myeloid Neoplasms

Secondary AML and t-MN are consistently associated with poorer prognosis when treated with standard induction chemotherapies [1,2,6,7,8,9,10,132]. Factors contributing to the poor survival include (i) treatment of the prior malignancy by selecting the chemoresistant clone; (ii) the acquisition of adverse-risk cytogenetic and molecular aberrations; (iii) the measurable persistence of disease-driving ‘secondary-type mutations’ during remission, as opposed to the later mutations (e.g., *NPM1*, *FLT3*), which are lost during remission; and (iv) older, less fit patients due to prior malignancy and/or therapies [6,8,10,11]. For this reason, many clinicians and researchers believe that classifying sAML and t-MN by pattern of genetic aberrations observed at diagnosis would be more relevant than the clinical classification, and guide therapeutic options to optimize chance of remission. Despite this, until recently, the treatment algorithms employed were not different from de novo AML.

A major breakthrough in the treatment of high-risk AML, including those arising from antecedent hematological malignancy and t-MN, is CPX-351. CPX-351 is a liposomal encapsulation of cytarabine/daunorubicin in a fixed 5:1 molar ratio. In a phase III trial of CPX-351 compared to standard 7 + 3 chemotherapy in 60–75-year-old newly diagnosed AML patients, CPX-351 led to a significantly higher remission rate (48% vs. 33%) and significant improvement in OS (9.6 vs. 6 months). A planned subgroup analysis showed favorable outcomes using CPX-351 in those with t-MN and AML secondary to MDS, which had not been treated, and AML arising from CMML, but not in those with treated MDS or de novo AML with an MDS-like karyotype [133,134]. CPX-351 is approved by the United States Food and Drug Administration (FDA) for treatment of all adults with newly diagnosed t-AML or AML-MRC, though it is worth noting that there is no evidence of improved outcomes in younger adults with these diseases.

B-cell lymphoma-2 (BCL-2) inhibitor venetoclax is a promising novel option for AML patients including high-risk AML subgroups. When combined with a hypomethylating agent (decitabine or azacitidine), it has been found to be highly effective in high-risk AML subgroups, including sAML (response rate 67%, same as the de novo cohort). Despite these very encouraging results, it is important to note that durable responses are uncommon (<25% at 1-year) and patients harboring *FLT3, RAS,* and *TP53* mutation have worse outcomes (response rate 50%, 33%, and 47%, respectively) [135].

Restoring wild type *TP53* function or blocking mutant *TP53* function would clearly be of great interest in *TP53*-mutated myeloid malignancies as *TP53* mutations are both common and predict exceedingly poor outcomes due to chemoresistant phenotype. Small-molecules p53 reactivation and induction of massive apoptosis (PRIMA)1 and its analog PRIMA-1^Met^ (APR-246) have been developed to target mutant *TP53* and restore transcriptional activity [136]. This drug, either as a monotherapy or in combination with other agents, is currently under investigation in myeloid malignancies in various phase I, II, and III trials (NCT03072043). Similarly, *PPM1D* is an attractive target for therapy. The cells harboring *PPM1D* mutations are chemoresistant and selectively expand in the presence of chemotherapy [110,137]. Treatment with a small molecular inhibitor of *PPM1D* reverses the chemo-resistance phenotype and preferentially kills *PPM1D*-mutant cells [137], representing a promising option for both prevention and treatment of *PPM1D*-mutated t-MNs.

Allogeneic stem cell transplant is recommended to patients with sAML and MPN-BP if patient is an appropriate candidate for intensive treatment; however, the role of transplant is more controversial in the case of t-MN [138]. A European registry study by Sengsayadeth et al. reviewed transplant outcomes for 4997 patients which included patients with sAML from MDS (65%), MPN-BP (15%), and t-MN arising from non-myeloid hematological malignancy (10%) or solid tumor (12%) who underwent allogeneic transplant [139]. OS at 2-years was 44%, though graft-vs-host-disease free relapse free survival (GRFS) at 2-years was low (27%). The study confirmed that patients with MPN-BP had inferior survival compared to those with sAML arising from MDS (30% vs. 40%). Lymphoma as the primary malignancy led to inferior survival (31%) compared to the rest of the cohort. Myeloablative conditioning was associated with a lower incidence of relapse, but a higher non-relapse mortality leading to no difference in OS compared to patients who received reduced-intensity conditioning. This study did not report on prognostic effect of molecular mutations on outcomes, but did demonstrate that active disease at time of transplant, adverse cytogenetics, age, poor performance status, and cytomegalovirus (CMV) positivity are associated with an inferior survival.

The role of allogeneic transplant remains controversial for *TP53*-mutated leukemia [140,141]. Practices vary dramatically among transplant centers—some do not recommend transplant to any patients with mutated *TP53*, some recommend transplant only if a complete remission (CR) is achieved, and others recommend transplant in the absence of another viable alternative. A Japanese bone marrow transplant registry study of high-risk MDS and sAML assessed relative contribution of *TP53* and CK status to post-transplant survival. Interestingly, *TP53* mutation, in the absence of CK, did not predict inferior survival, whereas those with CK had an abysmal prognosis irrespective of the *TP53* status [142]. It should be noted that the patients were relatively younger (median age at transplant: 53 years) and a higher proportion underwent myeloablative conditioning (65%), compared to comparable studies and day-to-day practice. In contrast, some studies have shown few or no long-term survivors [143,144,145,146]. Non-genetic factors—clinical characteristics, including age, performance status, disease status at transplant, and conditioning regimen—have all been purported to identify patients who may benefit from transplant.

In summary, while transplant results in long-term survival in some, survival rates are clearly suboptimal. Many patients, due to not having achieved remission, frailty, or the lack of donors, cannot undergo allogeneic SCT. Therefore, further research focusing on developing targeted strategies to achieve remission, identifying the optimal donor, conditioning regimen, identification of minimal residual disease pre- and post-transplant, and strategies such as post-transplant maintenance is urgently needed.

## 8. Conclusions

Transformative molecular technologies have led to a better understanding of driver mutations and unraveled subclonal architecture of secondary leukemia. It is clear that the current clinical classification (de novo AML, sAML, and t-MN) fails to capture the heterogeneity and complexity of these diseases. Efforts aimed at classifying these entities based on their genetic ontogeny are gaining momentum. A deeper insight into genetics will certainly be the key to improved prognostication and monitoring, as well as development of targeted therapeutics for these devastating diseases. As sequencing becomes more accessible, the next challenge will be to coalesce the massive amount of available information, including the identification of the genes involved, the functional characterization of mutations, and the impact of co-mutations, as well as the standardization of optimal VAF thresholds, and the integration of non-genetic factors, all culminating in meaningful and actionable clinical knowledge. Recent advances in non-genetic factors such as the bone marrow microenvironment, immune surveillance, and epigenetics will need to be integrated to derive truly personalized prognostic and therapeutic information for the patient [116,128,129]. The progress on the biological front of sAML and t-MN will need to be coupled with an increased awareness among clinicians, as well as a regulatory push to ensure that patients with secondary leukemia are enrolled in pertinent clinical trials.

## Figures and Tables

**Table 2:** Functional classification of the most commonly mutated genes in myeloid malignancies.

Epigenetic Regulators	RNA Splicing Factors	Transcriptional Regulator Genes	Activated Signaling Pathways
*TET2*	*SF3B1*	*RUNX1*	*CBL*
*IDH1/2*	*SRSF2*	*ETV6*	*NRAS*
*DNMT3A*	*U2AF1*	*IKZF1*	*KIT*
*EZH2*	*ZRSR2*	*CUX1*	*JAK2*
*ASXL1*		*TP53*	*MPL*
*SETBP1*		*PHF6*	*FLT3*
			*NF1*

**Table 3:** Mutations implicated in leukemic progression from myelodysplastic syndrome (MDS).

Functional Group	Gene	Location	Type of Mutation	Protein Function	Frequency in MDS (%)	Frequency in sAML (%)	HR for sAML	Ref.
Transcriptional regulators	*RUNX1*	21q22.3	Nonsense/missense/indel	Transcription factor in hematopoiesis	13	25–30	2.9	[16]
	*KMT2A*	11q23	Partial tandem duplication	Histone methyltransferase, transcription factor	4	14	3.1	[16]
	*TP53*	17p13.1	Missense/indel	Regulate cell cycle, DNA repair, apoptosis	10	15		[10]
Epigenetic regulators	*ASXL1*	20q11	Frameshift	Chromatin-binding associated w/PRC1/2	20	35	2.4	[47,48]
	*EZH2*	7q35-q36	Missense, indel	LOF H3K27 methyltransferase	4	9		[10]
	*IDH1*	2q34	Missense, hotspot	Enzyme, cellular protection from oxidative stress	5–10	11	7.0	[46,49]
	*IDH2*	15q26.1	Missense, hotspot		5	11	3.8	[50]
RNA splicing factors	*SRSF2*	17q25.1	Missense/hotspot	RNA splicing factor	15	20	2.8–3.9	[50]
Activated signaling pathways	*FLT3*	13q12	ITD	Cytokine receptor	<1	12–20	3.76	[16,46]
	*RAS*	multiple	Missense/activation	ERK/MAPK signaling	5	11–23	3.77	[16,46,51,52]
	*CSF3R*	1p34.3	Nonsense	Cytokine, controls the production, differentiation, and function of granulocytes	3	8	6.0	[50,53]

Abbreviations: myelodysplastic syndrome (MDS); secondary acute myeloid leukemia (sAML); hazard ratio (HR); insertion and/or deletion (indel); loss of function (LOF); internal tandem duplication (ITD).

**Table 4:** Mutations implicated in progression from MPN-BP [54].

Functional Group	Gene	Location	Type of Mutation	Protein Function	Frequency in MPN	Frequency in sAML (%)	HR of sAML	Ref
Activated signaling pathways	*FLT3*	13q12	FLT3-ITD	Cytokine receptor	<3% MPN	13		[36]
	*SH2B3*	12q24	Missense (LOF), deletion	Negative regulator of JAK2	1% ET; 2% PMF	13		[55,56]
	*CBL*	11q23.3	Missense (LOF)	Cytokine receptor internalization	4% PMF	8		[57,58]
	*NRAS*	1p13.2	Missense (activation)	ERK/MAPK signaling	Rare PMF	8	>2	[34,38]
	*NF1*	17q11	Missense deletion	ERK/MAPK signaling	Rare PMF	8		[59]
Epigenetic regulators	*TET2*	4q24	Missense, nonsense deletion	Active 5-methyl-cytosine demethylation	10–20% MPN	21	>2	[34,60]
	*DNMT3A*	2p23.3	Missense, hotspot	DNA methylase	5–10% MPN	18		[61]
	*IDH1*	2q34	Missense, hotspot	Enzyme, cellular protection from oxidative stress	<2% PV/ET 1–4% PMF	15–30	4	[32,62,63]
	*IHD2*	15q26.1	Missense, hotspot			15–30	2–55	[26,34,63]
	*EZH2*	7q35-36	Missense, indel	LOF H3K27 methyltransferase	3% PV 5–10% PMF	13	146	[26,41]
	*ASXL1*	20q11.1	Nonsense/indel	Chromatin-binding associated w/PRC1/2	1–3% ET/PV; 25% PMF	25	2	[32,64]
Transcriptional regulators	*TP53*	17p13.1	Missense/indel	Transcription factor, regulate cell cycle, DNA repair, apoptosis	<5% MPN	10–20	15–82	[26,34,37]
	*MDM4*	1q32.1	Amplification 1q	inhibits p53-mediated transcriptional activation	<1% MPN	18		[37]
	*CUX1*	7q22	Deletion 7q	Transcription factor regulating TP53 & ATM	<3% MPN	17		[39]
	*IKZF1*	7p12.2	Deletion 7p, indel	Transcription factor in lymphopoiesis	<3% MPN	10		[39]
	*RUNX1*	21q22.3	Nonsense/missense/indel	Transcription factor in hematopoiesis	<3% MPN	10–15	>2	[34,39]
RNA splicing	*SRSF2*	17q25.1	Missense, hotspot	RNA splicing factor	<2% ET; 15% PMF	15	3–74	[26,32,65]
	*U2AF1*	21q22.3	Missense	RNA splicing factor	10–15% PMF	13		[36,65]

Abbreviations: myeloproliferative neoplasm (MPN); secondary acute myeloid leukemia (sAML); primary myelofibrosis (PMF); polycythemia vera (PV); essential thrombocythemia (ET); hazard ratio (HR); insertion and/or deletion (indel); loss of function (LOF); internal tandem duplication (ITD); ataxia–telangiectasia mutated (ATM).
